# Cohort Profile: Project on Human Development in Chicago Neighborhoods and Its Additions (PHDCN+)

**DOI:** 10.1007/s40865-022-00203-0

**Published:** 2022-06-01

**Authors:** Robert J. Sampson, David S. Kirk, Rebecca Bucci

**Affiliations:** 1 Department of Sociology, Harvard University, Cambridge, MA USA; 2 Nuffield College and Department of Sociology, University of Oxford, Oxford, UK

**Keywords:** Life Course, Cohort, Criminal histories, Development, Chicago

## Abstract

The Project on Human Development in Chicago Neighborhoods (PHDCN) began in the mid-1990s, using an accelerated longitudinal design and drawing a representative sample of over 6200 children from a total of seven birth cohorts (ages 0 to 18) living in Chicago. Participants were followed for a second and third wave of data collection ending in 1998 and 2002, respectively. Independent surveys and observations on Chicago neighborhoods were also conducted. In 2012, a random subsample from cohorts 0, 9, 12, and 15 was selected for further follow-up, resulting in 1057 wave 4 interviews. In 2021, a fifth wave was launched to locate and survey wave 4 respondents, resulting in 682 responses. The extension to waves 4 and 5, termed the PHDCN+, is the main focus of this cohort profile. Survey data were collected from many domains including, but not limited to, family relationships, exposure to violence and guns, neighborhood context, self-reported crime, encounters with the police, attitudes toward the law, health, and civic engagement. In addition, official criminal records were collected for 1995–2020. The resulting PHDCN+ data includes five waves of comprehensive survey data, residential histories, neighborhood contextual data, and criminal histories extending over 25 years for four cohorts differing in age by up to 15 years. The research design, measures, key findings from the cohort sequential design, and data access opportunities are discussed.

## Why Was the Cohort Set Up?

The Project on Human Development in Chicago Neighborhoods (PHDCN) began in the mid-1990s as an interdisciplinary effort to unite the longitudinal study of individual lives with social context, especially neighborhoods, families, peers, schools, and the criminal justice system. Stemming from a partnership between the John D. and Catherine T. MacArthur Foundation and the National Institute of Justice (Tonry et al., 1991), the PHDCN was unusual in its cohort sequential design, enrolling over 6200 children from seven different birth cohorts ranging from infancy to age 18 in 1995, stratified by neighborhood socioeconomic status and race/ethnic composition. The resulting cohort populations were representative of the diversity of children in Chicago at the time and were studied over three waves of data collection, 1995–2002. The PHDCN was also notable for its detailed focus on the social, economic, organizational, political, and cultural environments in which crime and development take place, collecting original community surveys of Chicago residents and systematic social observations of the neighborhoods where the PHDCN children lived, again over the period 1995–2002.

To our knowledge, few if any prospective longitudinal studies in criminology have enrolled so many birth cohorts over such a wide age range, permitting the simultaneous analysis of both individual and social change. As Piquero et al. (2003, 410) noted in their review of the state of criminal career research, the design of the PHDCN “present[s] an unusual opportunity to examine period effects since successive cohorts will reach specific ages (e.g., age twelve) in different years and their life experiences can be compared.” Similarly, few if any studies have been designed to measure and study the role of neighborhood and other contextual environments in such a comprehensive fashion. The animating idea of PHDCN, therefore, was that by following multiple birth cohorts over the same historical time, and by independently assessing neighborhood social contexts, crime and the life course of human development could be studied in new ways.

From the outset, the PHDCN aimed to provide a public resource and, as such, was an early leader in the archiving of longitudinal data for public access at the University of Michigan’s National Archive of Criminal Justice Data, part of the Inter-University Consortium for Political and Social Research (ICPSR). Over 750 publications are listed on the project’s website, many appearing in leading journals and with the vast majority produced by researchers unaffiliated with the original project team.

The main funding from the MacArthur Foundation and the National Institute of Justice ended after the third wave of data, concluding the longitudinal study of the seven cohorts. Since then, however, a random sample of participants from four of the original PHDCN cohorts has continued to be followed and matched to other forms of contextual and criminal justice history information. The fourth and fifth waves of data collection took place in 2012–2013 and 2021, respectively, and criminal histories were collected through 2020, providing over 25 years of prospective longitudinal data on four of the original birth cohorts.

This cohort profile describes the full arc of what we label PHDCN+, with a focus on the more recent rounds of data collection and analyses that may be less familiar to the field.^1^ Key findings and innovations are emphasized, along with plans for future analyses. Data access information is also provided, continuing the legacy of the original study by serving as a public resource for developmental and life-course criminology, but also for the interdisciplinary and contextual longitudinal study of crime and human behavior more generally.

## Who Is In the Cohort?

The design of the PHDCN at baseline involved a two-stage procedure. First, a stratified representative sample of 80 neighborhood clusters was selected in the mid-1990s (out of 343 total), representing the wide variability, especially by race and class, of Chicago neighborhoods. A detailed array of data was collected from each neighborhood, including independent surveys of residents across the entire city of Chicago (*N* = 8872) and systematic observations of thousands of city streets in the 80 sampled neighborhood clusters. Second, for the main longitudinal study a representative sample of eligible children was drawn from a screening of more than 35,000 households in the 80 neighborhoods. Children falling within seven age cohorts at the time—infancy (born late 1994–1996), and then every 3 years until age 18 (i.e., age 3, 6, 9, 12, 15, and 18)—were then sampled from randomly selected households and studied over about 6 years, to the early 2000s.

Because of these procedures, the baseline PHDCN sample of just over 6200 was broadly representative of children and adolescents living in a wide range of Chicago neighborhoods in the mid-1990s. This was not a study just of the poor any more than it was a study just of those in trouble with the law. Chicago was selected not only because it is the nation’s third largest city and is broadly representative of the racial and ethnic diversity of the country, but also because it provided a rich source of institutional support and historical information to study the context of children’s lives in ways that could not be achieved in a national study.

## How Often Have They Been Followed Up?

### Waves 1–3

In the first round, or “wave,” of the study, collected between late 1994 and 1996, children were visited for extensive in-home interviews or assessments, along with interviews with their primary caregivers. Then, at roughly 2.5-year intervals, two more waves of data were collected by the PHDCN research team (wave 2 was concentrated in 1997–1999, and wave 3 in 1999–2001). Although all the children were living in Chicago at wave 1, and most stayed in Illinois or nearby (i.e., Indiana or Wisconsin), participants were followed no matter where they moved in the USA. Of the original PHDCN sample, which was recruited with a 75% overall participation rate at wave 1, 78% of participants took part in wave 3, response rates that are both relatively high for an urban sample. For sample sizes and response rates by cohort and wave, see https://www.icpsr.umich.edu/web/pages/NACJD/guides/phdcn/lsc.html#lsResponseRates.

### Wave 4

In 2011, Robert Sampson, one of the original Scientific Directors of PHDCN, launched a project with Robert Mare of UCLA that was funded by the MacArthur Foundation to locate and re-interview randomly sampled participants from four cohorts of the PHDCN. The data collection was carried out in 2012 and 2013. Of those last contacted at wave 3 of the PHDCN, a random subsample from the original infant cohort and the age 9, 12, and 15 cohorts was surveyed for an additional wave, here labeled “wave 5.” Resource constraints prohibited a follow-up of the full 6200+ respondents from the earlier waves of the original PHDCN, so careful consideration was given to which of the original seven cohorts to sample, as well as what proportion of the four sampled cohorts to include in the wave 4 data collection, in order to produce sufficiently powered analyses. The four cohorts sampled at wave 4 were selected to maximize variation in life course experiences and exposure to social change at different ages. Despite the long time that had elapsed since the last contact at wave 3—over a decade—and the difficulty of reaching people in an era of caller ID and increasing use of cell phones, 63% of eligible respondents took part overall (N=1057). Response rates varied from 61 to 67% by cohort status, with the youngest cohorts having the highest participation rate.

Ranging between ages 15 and 31 at wave 4, there are 378 respondents from the infant cohort, 227 respondents from the 9-year-old cohort, 235 from the 12-year-old cohort, and 217 from the 15-year-old cohort. The sample is nearly evenly split by gender (51% female, 49% male) and is diverse by race-ethnicity (19% white, 36.5% black, 40% Hispanic, and 4% Asian/other) and immigrant status (6% of cohort members were themselves 1st generation, 33% 2nd generation, and 61% 3rd generation). Like waves 1–3, detailed information was gathered from respondents in cohorts 9, 12, and 15, and from primary caregivers of the infant cohort as those respondents were just 15–17 years old at the time, on a wide array of topics. This included residential histories since wave 3, allowing investigators to merge census tract data on neighborhood conditions as well as crime rates to the survey data. The caretaker of the infant cohort member was also asked a battery of items that measured the behavior and circumstances of the infant cohort member as an adolescent, including aggression, antisocial behavior, low self-control, and depression.

### Wave 5

In 2021, NORC at the University of Chicago carried out a fifth wave of survey data collection funded by the National Collaborative on Gun Violence Research (NCGVR), directed by PIs Robert Sampson and David Kirk. Survey administration included an extensive effort to locate the 1057 wave 4 respondents. Whereas prior waves of the PHDCN were conducted in-person or over the telephone, the fifth wave added a web survey mode, with English and Spanish versions available for all modes. While the survey was not in the field during the height of Covid-19-related lockdowns, in the context of the pandemic the web mode proved important for securing respondents’ participation. Data collection began in May 2021 and closed on October 27, 2021.

Over the course of the data collection, we discovered that 16 of the respondents from wave 4 were ineligible to participate because they were deceased and one was incarcerated for the entirety of data collection and was unable to be interviewed.^2^ Of the remaining 1040 individuals, 682 completed the survey, with the vast majority on the web (71%), followed by phone (24%) and a small percentage were completed in-person (5%). We note, however, that both the phone and in-person contacts often led to a survey being completed on the web, suggesting the advantages of a multi-mode design. 

The final respondents at wave 5 range between ages 24 and 41, with 135 respondents in the 9-year-old cohort, 165 in the 12-year-old cohort, and 165 in the 15-year-old cohort. There are 217 children in the infant cohort who were on average 26 years old at wave 5. Like wave 4, wave 5 is diverse by race/ethnicity, with 21% white, 32% black, 42% Hispanic, and the remaining Asian or other. Table 1 provides the wave 5 sample by key characteristics and corresponding response rates.

**Table 1:** Key wave 5 characteristics and response rates

	Composition of completed sample	Response rates of eligible sample
Cohort		
0	31.8%	57.7%
9	19.8%	61.4%
12	24.2%	71.7%
15	24.2%	77.1%
Gender		
Male	45.5%	61.4%
Female	54.5%	69.5%
Race		
NH White	21.0%	71.9%
NH Black	32.3%	58.8%
Hispanic	42.2%	68.1%
Other	4.5%	70.5%
Residence		
Chicago	51.2%	63.2%
Illinois (non-Chicago)	28.4%	74.9%
Outside Illinois	20.4%	60.7%
Parental immigrant status		
First generation	38.9%	70.1%
Second generation	9.4%	66.7%
Third generation or higher	50.9%	62.7%
	N = 682	65.6% (N =1040)

**Fig. 1:**
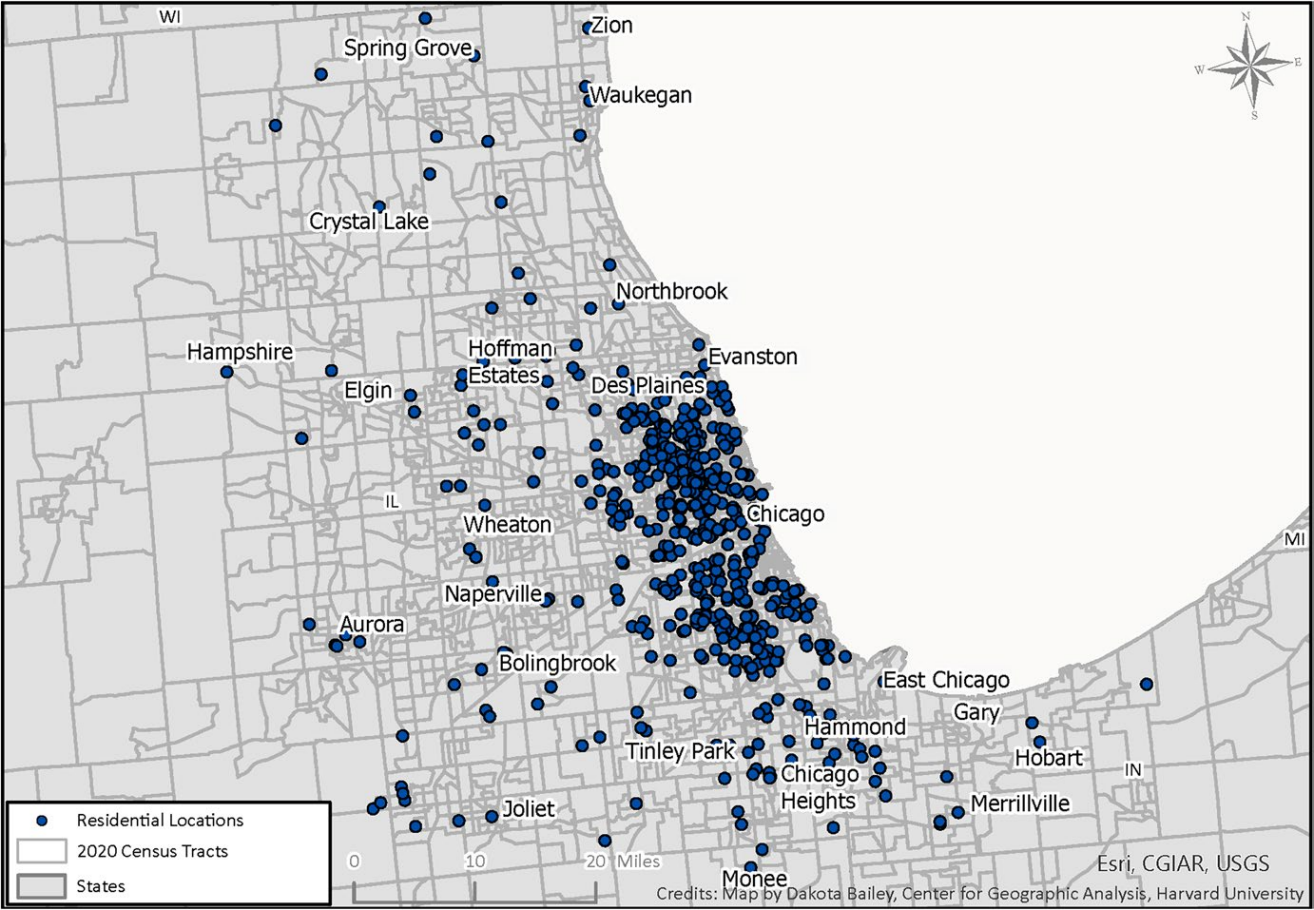
Location of PHDCN+ respondents in Chicago Metropolitan Area, 2021

**Fig. 2:**
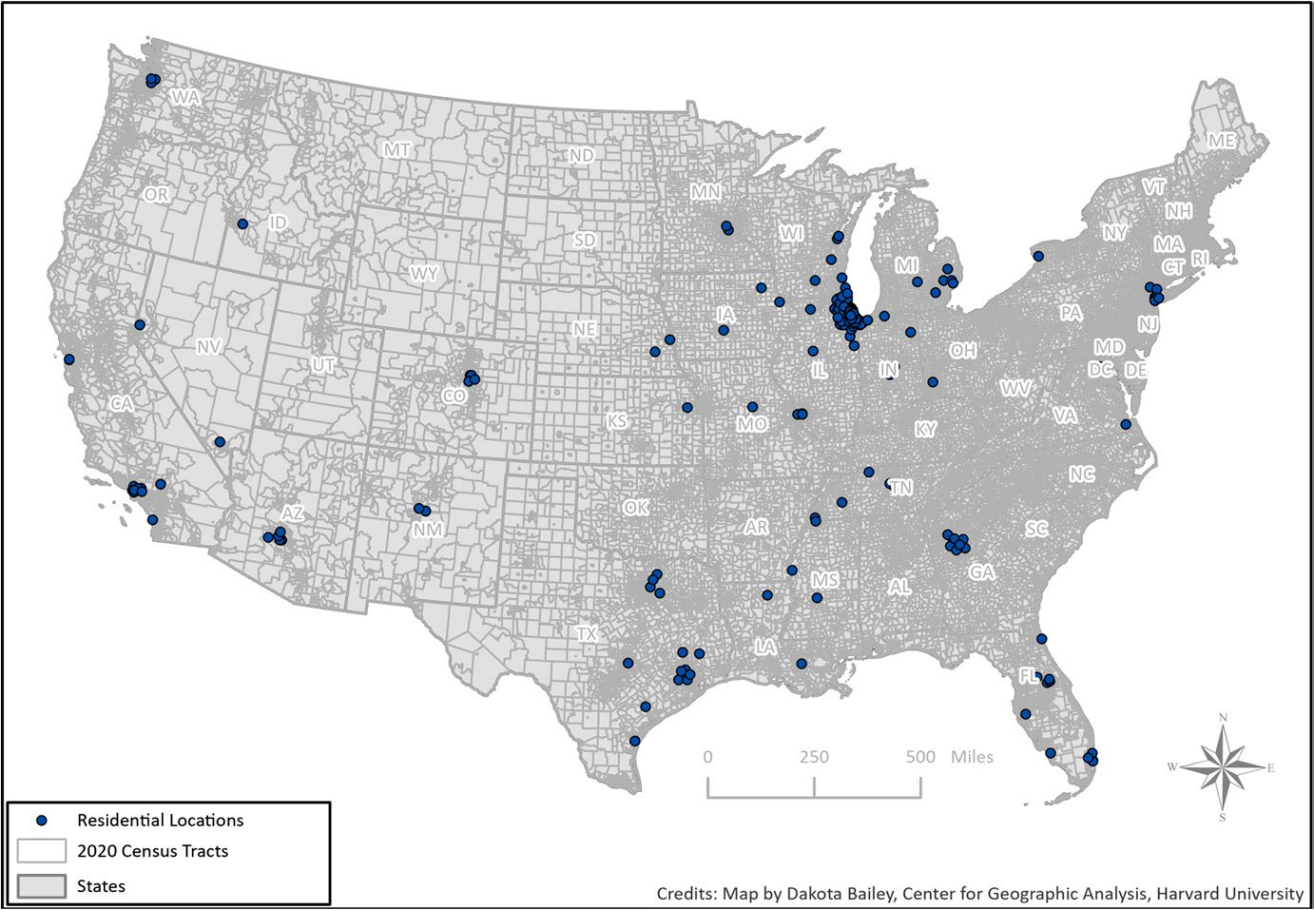
Location of PHDCN+ respondents nationally, 2021

Recall that at the study inception, the sample included a representative mix of Chicago youth from a stratified sample of neighborhoods. As shown in Table 1, 51% of sample members who completed wave 5 lived in the city of Chicago in 2021, with another 28% outside of the city limits but still within the state of Illinois. Forty-two percent of the wave 5 respondents lived in the city of Chicago at each survey wave, with the remaining sample members including those who left Chicago and have not returned, as well as respondents who have moved in and out of Chicago across the study periods. Figure 1 shows the distribution of respondents in the Chicago area, indicating a spreading out of respondents from the original 80 neighborhoods to the north, west, and south (see Sampson, 2012, 80). Figure 2 shows the distribution across the entire country.^3^ Although most people remain in Illinois or nearby, such as various cities and towns in Indiana, there is representation across the country, with clusters in Phoenix, New York, Los Angeles, and many areas in the South.

### Attrition

At present, we calculate the response for wave 5 to be 65.6%. This figure may change slightly as further information is obtained on eligibility status in 2021 from death record and criminal history searches. The wave 5 response rates ranged from 57.7% for the original infant cohort to 77.1% for the age 15 cohort (Table 1). Females were more likely to respond (69.5%) compared to males (61.4%), and non-Hispanic whites were the most likely to complete the survey (71.9%) while non-Hispanic blacks were the least likely (58.8%). Of those presumed to be living in Chicago, 63.2% completed the survey while 74.9% of those presumed to be living in Illinois but outside Chicago completed the survey. The lowest response rate was for those living outside Illinois (60.7%). Response rates were also higher among those born to first-generation immigrants (70.1%) compared to those respondents born to second and third-generation immigrants (66.7% and 62.7%, respectively).

While overall attrition was low for a contemporary urban sample and cohort differences are small, differential response rates over time do leave open the possibility of nonrandom attrition that could affect analyses. We therefore plan to conduct analyses similar to those conducted in earlier waves to simultaneously account for attrition and features of the survey design. Sampling weights at baseline adjust for the original stratification of the PHDCN by neighborhood SES and racial composition, along with the age cohort selection and a post-stratification of population weights to estimates of the age, gender, and race/ethnicity distribution of children in Chicago in 1995. With respect to loss due to follow-up, attrition weights were calculated by first estimating a model for the probability of attrition by wave that included indicators of the primary caregiver’s gender, citizenship status, age, SES, home ownership, household size, marital status, and social ties; the subject’s gender, race/ethnicity, immigrant generation, and age; and neighborhood characteristics, including racial and socioeconomic composition and collective efficacy. Based on these models, attrition weights were calculated by taking the inverse of each subject’s probability of response and standardizing these values by dividing by the mean.^4^ Analysis of weighted and unweighted data has also produced similar results in longitudinal analyses of criminal histories (Neil & Sampson, 2021).

As noted, at wave 5, approximately 66% of eligible respondents completed the survey. Preliminary attrition analyses suggest that in addition to differential rates by cohort, gender, race/ethnicity and residency, attrition was higher among those with an official arrest record from ages 17 to 24. Attrition is also more likely for those with lower educational attainment. Initial wave 5 sampling weights constructed by NORC address the nonrandom attrition by race/ethnicity and cohort. We plan to conduct new attrition analyses that include these and other predictors of wave 5 attrition and capitalize on the existing longitudinal data to best address survey nonresponse.

## What Has Been Measured?

The PHDCN was designed to advance the study of the developmental pathways of both positive and negative human social behaviors. Most relevant to developmental and life-course criminology, published papers on the data have examined the pathways to juvenile delinquency, adult crime, substance abuse, and violence. At the same time, the project also provided a detailed look at the environments in which these social behaviors take place by collecting substantial amounts of data about urban Chicago, including its people, institutions, and resources. As such, the measurement scheme for the PHDCN waves 1–3 varied by cohort, substantive domain, wave of data collection, and unit of analysis (e.g., child, caretaker, family, neighborhood). Hundreds of measurement instruments were administered, and full details can be found here: https://www.icpsr.umich.edu//web/pages/NACJD/guides/phdcn/lci.htm.

An advantage of the larger PHDCN+ data is the rich information available on individuals, their childhood, their families, their early-life neighborhood contexts, and their experiences in adolescence and young adulthood. When combined with the criminal justice data, the PHDCN+ thus provides an unusual opportunity for analyzing criminal trajectories. Neil and Sampson (2021) identified seven classes of factors in their analysis of criminal trajectories that provide a sense of the breadth and depth of measures. These include *demographics*, *family structure and background*, *exposure to crime and violence*, *neighborhood structure and social processes*, *family troubles*, *childhood behavioral troubles*, and *time-varying turning points*. For example, in addition to basic demographic variables, there is comprehensive family and household information such as parental age at birth of child, parental employment, public assistance, education, income, marital status, residential stability, household size, and homeownership. Beyond structural features, multiple dimensions of family processes were measured, such as parental supervision, warmth, parental verbal ability, social support, religiosity, parent–child conflict, and general family functioning measured by the Home Observation for Measurement of the Environment (HOME) instrument. Similarly, the PHDCN has a strong set of measures on self-reported delinquency and police contact and exposure to violence at the individual, family, and neighborhood level (e.g., Bingenheimer et al., 2005; Sampson & Sharkey, 2008; Sharkey, 2010). Because of the strong ties between direct and indirect exposure to violence and criminal justice contact (Western, 2018), the inclusion of this set of factors ameliorates a source of omitted variable bias common to many studies.

By design, the PHDCN+ has theoretically relevant *neighborhood characteristics* that go beyond traditional census factors. One is neighborhood lead levels measured in the blood of children who reside there (Sampson & Winter, 2018). Lead exposure can lead to crime, and children’s exposure to it has varied dramatically in recent decades (Muller et al., 2018). The second is a key neighborhood social process shown to predict crime—collective efficacy (Sampson et al., 1997). The third is crime and violence. Crime rates began to plummet in Chicago just as the youngest cohorts were born, meaning that exposure to violence, and to its consequences, varied markedly across cohorts. We are currently matching violent crime rates in Chicago over the full period of PHDCN+ to each respondent. The data also include both objective and perceived measures of physical and social disorder, as well as the extent of alcohol stores and secured buildings, determined through videotaped systematic observations and an “ecometric” measurement strategy (Raudenbush & Sampson, 1999). As many publications using these data have shown, the inclusion of these measures represents a significant advantage in terms of predicting crime and criminal justice contact both in terms of model fit and bias reduction (e.g., Kirk, 2009; Kirk & Matsuda, 2011; Morenoff et al., 2001; Sampson & Raudenbush, 1999; Sampson et al., 1997, 2005, 2008).

On *family troubles*, the study includes an array of problems that may beset families, which may impact children’s subsequent life course of crime, and which may vary by cohort and predict intergenerational problems. These include the extent to which family members have had trouble with the police, were incarcerated, had trouble with alcohol, and had drug problems, as well as parents’ exposure to violence, and whether they suffered from anxiety and depression. These are “criminally relevant” differences at the parental or family level and which varied across cohorts during a period that saw the rise of mass incarceration, the fall in violence, and the rise and fall of the crack epidemic, among other changes.

There are also a wide variety of measures of *childhood behavioral problems*. A longstanding view in developmental and life-course criminology is that early-life behavioral problems set in motion a cycle of events that perpetuate persistent behavioral problems, in addition to tapping individual propensities that predict later criminality (Farrington, 1998; Gottfredson & Hirschi, 1990). Moffitt (1993) also posits the importance of personality traits for the understanding of criminal trajectories. To account for and test these theories, measures include a multi-construct temperament or personality scale, self-control, antisocial behavior, aggression, peer delinquency, truancy, and grade retention—all mainstays of delinquency theory. Neil and Sampson (2021), for example, measured antisocial behavior or aggression, impulsivity, and anxiety/depression from the Child Behavior Checklist (CBCL), a widely used, reliable, and valid reporting measure for identifying emotional and behavioral problems (Achenbach, 1997). Other measures of internalizing disorders exist, as well as self-reported delinquency (by crime type), substance use, and criminal justice contact.

Finally, drawing on the age-graded theory of Sampson and Laub (1993), and its extensions (Laub & Sampson, 2020; Laub et al., 2019), there are measures for a range of *time-varying turning points* at waves 1-3 for the older cohorts with respect to domains of employment (e.g., unemployment or under-employment), marital status (e.g., single, married, cohabitating, divorced/separated), parental status, and residential mobility. The wave 4 and 5 interviews also have a battery of questions on recent jobs, current occupation, marital status, income, and residential locations for all cohorts. Of course, criminal justice contacts can also be a negative turning point, as indicated in research examining the effects of juvenile arrest on later outcomes such as education and further arrest (Kirk & Sampson, 2013; Liberman et al., 2014).

### New Data and Measures

Whereas much of our discussion of measurement thus far has described measures from waves 1 to 3, below we highlight new additions to the PHDCN+ repository following collection of wave 4 and 5 survey data as well as official criminal records.

In the waves 4 and 5 surveys, particular emphasis was directed toward collecting information on the following measurement domains:

- Residential histories
- Perceptions of residential neighborhoods
- Social relations
- Civic and political engagement
- Victimization
- Access to and use of guns
- Self-reported offending
- Views of the law and the police
- Criminal justice contacts and experiences with the police
- Family structure and parenthood
- Housing and living arrangements
- Education and employment
- Earnings, assets, and debts
- Mental health and physical health, including Covid-19.

In addition to the extensive survey data collected at waves 4 and 5, information on respondents’ criminal histories was collected over this period. Arrest and subsequent criminal records starting in 1995 were collected from the Criminal History Record Information (CHRI) in Illinois and analyzed. The criminal history records cover all jurisdictions in Illinois, including small town police departments and county probation offices, and include detailed information, by date, on arrests, charges, dispositions, and sentences, including fines, probation type, jail, and prison. Wave 4 (and by extension, wave 5) respondents were matched by name and date of birth with CHRI records covering the entire state of Illinois four times, in 2015, 2017, 2019, and early 2021, measuring the sequencing of arrests by age from 1995 through calendar year 2020. A total of 381 of the 1057 PHDCN+ members from waves 1–4 were arrested (36%), generating 2739 charges and 1721 arrests—and the youngest birth cohort has only just now reached its mid-twenties, a prime age of turning points and first incarceration. There are also data on nearly 700 sentences for 172 individuals. Given the rich interview-based information, the data allow analysis of the prediction and explanation of official criminal histories over the life course in a cohort sequential design spanning more than a quarter-century.

## What Has It Found? Key Findings and Publications

As noted, there have been hundreds of studies published on the PHDCN, many by scholars unaffiliated with the original project. Some of the more well-known findings from the use of the cohort data from waves 1 to 3 include a host of studies examining the near-term consequences for youths of growing up in socially disadvantaged and violent neighborhoods. For instance, Sampson et al. (2005) dissected reasons for racial and ethnic disparities in violent offending, finding that a large source of difference is due to the fact that race-ethnic groups tend to reside in fundamentally different neighborhood contexts. Following a similar theme, Sampson et al. (2008) find that residence in severely disadvantaged neighborhoods is detrimental to the development of verbal ability, such that residing in neighborhoods marked by concentrated disadvantage between waves 1 and 2 of the PHDCN had a similar impact on verbal ability for Black youths as missing an entire year of schooling.

Among the many PHDCN studies of the causes and consequences of exposure to violence, Bingenheimer et al. (2005) find that exposure to firearm violence doubles the probability than an adolescent would engage in serious violence over the ensuing two-year period. Sharkey and Sampson (2010) find that whereas neighborhood moves within Chicago lead to an increased risk of violence, moves outside the city reduce violent offending and exposure to violence. The gap in violence between movers within and outside Chicago is explained not only by the racial and economic composition of the destination neighborhoods but also by the quality of school contexts, adolescents’ perceived control over their new environment, and fear. And consistent with the neighborhood focus of PHDCN and building on the theoretical and empirical work on collective efficacy and legal cynicism using the Community Survey component of the study (Kirk & Papachristos, 2011; Sampson & Bartusch, 1998; Sampson et al., 1997), Kirk and Matsuda (2011) analyze both the Community Survey and early cohort data and find that cynicism of the law undermines neighborhood collective efficacy, thereby disentangling one reason why violence proliferates in neighborhoods with high levels of legal cynicism.

However, only 6 years of development were available for analysis in the first three waves of the PHDCN, restricting the kinds of life-course analyses envisioned by the original designers of the study. With the recent waves of data collection and criminal histories, long-term analyses of crime, human development, and the life course are now possible, including an assessment of the cumulative consequences of exposure to certain types of social environments. Analyses of the wave 4 data linked to waves 1–3 and administrative data, such as neighborhood census tract data and criminal records, have yielded papers on a wide range of topics, finding, for example, the cumulative effects of neighborhood disadvantage on reduced educational attainment (Levy et al., 2019), racial inequality in trajectories of compounded disadvantage (Perkins & Sampson, 2015), and the deleterious consequences of exposure to lead for later delinquent and other problem behaviors (Sampson & Winter, 2018; Winter & Sampson, 2017).

A central idea of the life course paradigm as articulated by Elder (1985, 1994) is the embeddedness of individual development in social contexts that change through time and across place. Laub and Sampson (2020) argued that linking individual and social change through cohort analysis is a frontier issue for life-course and developmental criminology. Motivated by this charge, Neil and Sampson (2021) advanced and tested hypotheses on arrest in the lives of 1057 individuals from wave 4 whose criminal histories were collected from 1995 through 2018. The PHDCN + cohort sequential design, combined with the multiple stages of data collection and rich measurement, allowed the authors to examine life-course trajectories during a time of rapid social change, including both intra- and inter-cohort variations.

Neil and Sampson’s (2021) results reveal how social change altered the experience of criminal justice contact in adolescence and early adulthood in meaningful ways. The probability of being arrested was nearly twice as large during the peak ages of delinquency in adolescence for cohorts born in the early to mid-1980s compared to younger cohorts born in the mid-1990s, and there was a much faster rate of decline in the probability of arrest in early adulthood for the younger birth cohorts. These findings were not driven by differences between birth cohorts in alternative explanations such as individual dispositions, demographic and family background, economic status, or early-life neighborhood environments, several of which were large. Rather, the substantial cohort differentials in arrest in late adolescence and the course of desistance in adulthood stemmed from the distinct socio-historical environments through which each cohort aged. The authors also found that the impact of family socioeconomic disadvantage and individual self-control on arrest varied by cohort status. Sampson and Smith (2021, 51–54) extended these analyses to include criminal histories through 2020, a period that included the large rise in violence during 2019 and 2020, finding similar patterns of cohort differentiation. These studies thus reveal the power of social change to influence patterns of criminality.

In another paper, Neil et al. (2021) revisit the classic finding of Wolfgang et al. (1972) that animated nearly 50 years’ worth of research on chronic offenders and other types of offending groups, such as life-course persistent and adolescent-limited offenders (Moffitt, 1993). They posed two questions. First, are there distinct trajectories of offender-group membership, defined by arrests, and if so, do they vary by cohort? Second, do cohort differences in offender-group membership reflect the fact that cohorts differ demographically or in their level of exposure to risk factors? Establishing whether cohort differences reflect the dynamic influence of the social environment across the life course is crucial in interpreting what cohort differences represent and what that means for our understanding of offending groups more broadly in terms of theory and policy.

The results in Neil et al. (2021) are consistent with past research in revealing three basic offender groups—one with few or no arrests over the life course, a second group of “chronic” or “life-course persistent” patterns of arrest, and a third “middle-ground” group that peaks in adolescence and declines but at a much lower rate, similar to what Moffitt (1993) called “adolescent-limited” offenders. Whatever label we assign to each group, estimated membership depends on when each cohort came of age. For example, controlling for a wide-ranging set of demographic characteristics and early-life risk factors similar to those employed in Neil and Sampson (2021), the older cohorts had an odds of membership in the Medium arrest trajectory group compared to the Low group over five times higher than the younger cohorts. The odds of membership in the High “chronic” group as opposed to the Low group was over 2.5 for the older compared to the younger cohorts, and both differences were significant (*p* < 0.05). Therefore, not only does cohort status predict group membership independent of demographic differences and early risk factors, but cohort differentiation through social change is comparable in size to the influence of several notable risk factors, such as of poverty and self-control.

Future analyses will examine a number of topics, with a major focus on how chronic exposure to gun violence is associated with mental and physical health and well-being, and if early life exposure to gun violence continues to have a lasting impact on the life course even if the exposure to such conditions ceases. We will also examine the life course of legal cynicism by assessing how childhood and adolescent characteristics and environmental conditions are associated with the development of cynical views of the law. Similarly, we will explore how perceptions of collective efficacy change over the life course, as neighborhoods change, and people move. Leveraging both the survey data and official criminal histories, another major focus of analysis will be the intergenerational transmission of criminal justice contact.

Finally, we will continue to test for cohort differentiation in these and other issues. Through wave 5, the PHDCN+ data collection has taken place over a 26-year period that included the great crime decline, the rise and flattening of mass incarceration, the loosening of gun laws, large fluctuations in police practices, the Great Recession and foreclosure crisis, the establishment and advance of the Black Lives Matter movement, the Covid-19 pandemic, the 2021 Capitol Insurrection, and large increases in violence from 2019-2021. Cohort differences in aging through this period of substantial and varied social change have important but largely unstudied consequences for crime, gun violence, and life-course development, and our next phase of PHDCN+ studies will emphasize such questions. For instance, future analyses will examine cohort differences in exposure to violence over the past three decades, particularly gun violence. We will also examine how the risk factors of gun violence may have evolved over time with respect to societal change.

## What Are the Main Strengths and Weaknesses?

Summarizing the strengths described in earlier sections, the PHDCN+ is characterized by:

- A sequential and overlapping cohort design that began with seven different birth cohorts over three waves, ages 0, 3, 6, 9, 12, 15, and 18 at baseline in 1995.
- Five waves of longitudinal data spanning 26 years for four cohorts (ages 0, 9, 12, and 15 at baseline).
- A distinct focus on the social, economic, organizational, political, and cultural environments in which crime and development take place, via data collected from independent surveys of neighborhood residents and systematic social observations of neighborhood conditions in addition to longitudinal cohort data.
- A merging of administrative data to cohort survey data, including three decades of official criminal records as well as Chicago Public Schools educational records.

The PHDCN+ still has limitations, of course. It is based on cohorts originally from Chicago, raising generalizability questions. We would note, however, that many of the major longitudinal studies in criminology are based on specific cities, such as Pittsburgh, Rochester, Toledo, Denver, Phoenix, Philadelphia, London, Montreal, and Zurich, among others. Rich data collection constrained by the limits of grant funding almost demand a focus on single cities. That said, the PHDCN+ followed people wherever they moved in the USA (Fig. 2).

Another potential limitation is the exclusion of three of the original seven cohorts during the waves 4 and 5 data collection. As discussed earlier, resource constraints necessitated that investigators conduct a subsample of the original waves 1 to 3 respondents, thereby excluding the age 3, 6, and 18 cohorts. Nevertheless, the four cohorts sampled at wave 4 were specifically selected to maximize variation in life course experiences and exposure to social change at different ages. Similarly, it was not possible to include all measures collected in waves 1–3 at waves 4 and 5 due to both resource constraints and concerns over the survey completion time.

A limitation of the criminal history data is that, at present, we are restricted to criminal histories in the state of Illinois. It is possible that criminal histories are underestimated if respondents commit crimes in adjacent jurisdictions or if respondents no longer live in or near Illinois but are criminally active. However, the proportion of the sample that still lived in Illinois at wave 4 was substantial (88%). Others lived in Illinois for many years just up to wave 4, while some moved out in earlier waves and then moved back after wave 4. Only 2% of the sample moved out of Illinois at wave 2 and never returned; these cases have been excluded in prior analyses of the criminal history data. Of those who completed the wave 5 survey, 543 (80%) currently live in Illinois. Of the 358 eligible non-respondents, it is estimated that 254 remain in Illinois, with 179 of those eligible non-respondents remaining in Chicago, translating to approximately 77% of the eligible 1040 cases currently or recently living in Illinois.

Attrition is a concern in longitudinal studies conducted over an extended period of time, and the PHDCN+ is no exception. As a result, careful attention has been paid to the predictors of attrition. Given the breadth of data for each respondent, comprehensive attrition and sampling weights will be used in future analyses, where relevant, and compared to unweighted analyses.

A consequence of the decision at waves 4–5 to sample from only four of the original study cohorts is that the combined sample size for PHDCN+ analyses is more limited than the original study design, with potential implications for statistical power. Nevertheless, we have at least 135 respondents for each of our four cohorts, thereby permitting well-powered analyses.

## Can I Get Hold of the Data? Where Can I Find Out More?

The first three waves of the longitudinal cohort data, along with the 1995 Community Survey and Systematic Social Observations, are presently archived at ICPSR (https://www.icpsr.umich.edu//web/pages/NACJD/guides/phdcn/index.html). Waves 4–5 and other PHDCN+ data will be made publicly available after the close of currently funded grants. For potential collaborative research, contact the corresponding authors.

## Profile in a Nutshell

- Rationale: The PHDCN+ is a continuation of a multi-cohort interdisciplinary longitudinal study that began in the mid-1990s that was designed to unite the longitudinal study of individual lives with social context, especially neighborhoods, families, peers, schools, and the criminal justice system. The full data collection consists of five survey waves spanning 26 years. The two most recent waves, from 2012 and 2021, allow for the continued examination of four cohorts differing in age by up to 15 years. The data tied to the existing intergenerational data on family and social contexts, as well as criminal justice system exposure, is a rich resource to examine social change and individual-level differences, including the correlates, predictors, and outcomes related to criminal justice involvement and gun violence.
- Sample: The original PHDCN sample consists of over 6200 respondents who were followed over three waves, 1995–2002. Respondents are a representative sample of children from a stratified sample of Chicago neighborhoods. The PHDCN+ subsample consists of 1057 of the original respondents measured across four survey waves (1995–2012, from four birth cohorts), with a fifth survey wave completed by 682 of these respondents in 2021. As with previous waves, wave 5 respondents are diverse by race/ethnicity, with 21% white, 32% black, 42% Hispanic, and the remaining Asian or other.
- Follow-ups: Of the original wave 1 to 3 respondents from the four cohorts selected for additional follow-up, 378 of the infant cohort, and 227, 235, and 217 of the 9, 12, and 15 cohorts completed the wave 4 survey, respectively (*N* = 1057). At wave 5, 217 members of the infant cohort completed the survey, as did 135, 165, and 165 of the 9, 12, and 15 cohorts, respectively (*N* = 682).
- Measures: Wave 1 to 3 data include an extensive battery of information about child and adolescent development, with wave 4 and 5 data tapping a broad array of measures about early and mid-adulthood, including information on residential histories, perceptions of neighborhoods, victimization, firearm access and use, offending, views of the law and the police, and criminal justice contact.
- Data access: The first three waves of the longitudinal cohort data are archived at ICPSR: https://www.icpsr.umich.edu//web/pages/NACJD/guides/phdcn/index.html. PHDCN+ data will be made publicly and freely available in 2024.

## Footnotes

1 A comprehensive discussion of the project’s original motivation, intellectual history, investigative team, research design, and data collection details on the first three waves can be found in Sampson (2012, 77–93).

2 Given the difficulties in contacting and interviewing currently incarcerated or otherwise incapacitated persons (e.g., those in the hospital or with serious injuries), sampled respondents who were currently incarcerated or incapacitated were considered out of scope at waves 4 and 5. Seventeen individuals were incarcerated at the time of the wave 4 interview.

3 Two additional wave 5 respondents live outside of the US and are not represented in Figure 2.

4 Official juvenile arrests of the participants through wave 3 were also included as a predictor to account for potential bias in follow-up at wave 4 by criminality. Although criminalized men are often among the hardest to reach, the conditional probability of follow-up at wave 4 was only marginally different among those who had and had not been arrested in their youth. A combined wave 4 weight was created by multiplying PHDCN’s initial survey design by attrition weights for waves 3 and 4.
